# Suicide and Other-Cause Mortality after Early Exposure to Smoking and Second Hand Smoking: A 12-Year Population-Based Follow-Up Study

**DOI:** 10.1371/journal.pone.0130044

**Published:** 2015-07-29

**Authors:** Vincent Chin-Hung Chen, Chian-Jue Kuo, Tsu-Nai Wang, Wen-Chung Lee, Wei J. Chen, Cleusa P. Ferri, Duujian Tsai, Te-Jen Lai, Meng-Chuan Huang, Robert Stewart, Ying-Chin Ko

**Affiliations:** 1 Chang Gung Medical Foundation, Chiayi Chang Gung Memorial Hospital and Chang Gung University, Chiayi, Taiwan; 2 Department of Psychiatry, Chung San Medical University Hospital, Taichung, Taiwan; 3 School of Medicine, Chung Shan Medical University, Taichung, Taiwan; 4 Taipei City Psychiatric Center, Taipei City Hospital, Taipei, Taiwan; 5 Department of Psychiatry, School of Medicine, Taipei Medical University, Taipei, Taiwan; 6 Department of Public Health, College of Health Science, Kaohsiung Medical University, Kaohsiung, Taiwan; 7 Institue of Epidemiology and Preventive Medicine, College of Public Health, National Taiwan University, Taipei, Taiwan; 8 Universidade Federal de São Paulo-Psychobiology Department, São Paulo, Brazil; 9 School of Medicine, Taipei Medical University, Taipei, Taiwan; 10 Institute of Medicine, Chung Shan Medical University, Taichung, Taiwan; 11 Department of Public Health and Environmental Medicine, School of Medicine, College of Medicine, Kaohsiung Medical University, Kaohsiung, Taiwan; 12 Department of Nutrition and Dietetics, Kaohsiung Medical University Hospital, Kaohsiung, Taiwan; 13 King’s College London (Institute of Psychiatry), London, United Kingdom; 14 Environment-Omics-Diseases Research Center, China Medical University Hospital, Taichung, Taiwan; 15 Graduate Institute of Clinical Medical Science, China Medical University, Taichung, Taiwan; Chiba University Center for Forensic Mental Health, JAPAN

## Abstract

**Background:**

The association between smoking and suicide is still controversial, particular for early life cigarette smoking exposure. Few studies have investigated this association in adolescents using population-based cohorts, and the relationship with second hand smoking (SHS) exposure has not been addressed.

**Methods and Findings:**

In this study, we followed a large population-based sample of younger people to investigate the association between smoking, SHS exposure and suicide mortality. Between October 1995 and June 1996, 162,682 junior high school students ages 11 to 16 years old living in a geographic catchment area in Taiwan were enrolled and then followed till December 2007 (1,948,432 person-years) through linkage to the National Death Certification System. Participants who were currently smoking at baseline had a greater than six-fold higher suicide mortality than those who did not smoke (29.5 vs. 4.8 per 100,000 person-years, p<0.001) as well as higher natural mortality (33.7 vs. 10.3 per 100,000 person-years, p<0.001). After controlling for gender, age, parental education, asthma, allergic rhinitis, and alcohol consumption, the adjusted hazard ratios for suicide were 3.69 (95% CI 1.85-7.39) in current smokers, and 1.47 (95% CI 0.94-2.30) and 2.83 (95% CI 1.54-5.20) respectively in adolescents exposed to SHS of 1-20 cigarettes and >20 cigarettes/per day. The estimated depression-adjusted odds ratio did not change substantially. The population attributable fractions for suicide associated with smoking and heavy SHS exposure (>20 cigarettes/per day) were 9.6% and 10.6%, respectively.

**Conclusions:**

This study showed evidence of excess suicide mortality among young adults exposed to active or passive early life cigarette smoking.

## Introduction

Adolescent smoking is a pressing public health issue especially in areas of rapid economic growth, and more than 80% of adult smokers have their first experience of smoking before the age of 18 [1]. A disproportionately high percentage of the world’s smokers are in East Asian countries, and 82% of the approximately 1.1 billion smokers worldwide reside in developing countries [2]. The global mortality attributable to smoking is estimated at over 6 million annually, with nearly two-thirds of these deaths occurring in developing countries [3].

As well as all-cause mortality, some prospective studies have also shown a significant association between smoking and suicide in adults [4]. Explanations for this association include pre-existing conditions in smokers increasing their risk for suicide (i.e. confounding), debilitating conditions secondary to smoking that might lead to suicide (i.e. causal pathway factors), and effects of smoking to decrease serotonin and monoamine oxidase levels [5]. However, previous studies have been limited. Many have not controlled for characteristics common to both smokers and persons who commit suicide [6], and few have used nationally representative samples or prospective designs [6–9]. A previous study carried out in Taiwan failed to find an association between smoking and suicide [10], but few studies have investigated this in adolescents [4], although adolescence is a key period of importance for smoking initiation [1], and one cohort study found a higher risk of suicide in adolescents who were smoking [11]. Potential consequences of second hand smoking (SHS) also deserve attention. Adverse physical health conditions such as persistent respiratory symptoms [12] in adolescents result from SHS and have been found to be associated with psychological distress and risk of future mental disorder in adulthood [13]. Among non-smoking children and adolescents aged 8 to 15 years with SHS exposure, serum cotinine levels were found to be positively associated with major depressive disorder, generalized anxiety disorder, attention-deficit/hyperactivity disorder and conduct disorder [14]. Since all above mental disorders are linked to suicide, the relationship between SHS and suicide warrants further attention. However, no research to date has investigated suicide as an outcome for SHS.

The prospective analysis described here investigated the smoking-suicide connection in a young population taking into account a range of potential confounders. Secondhand smoke was also investigated as an exposure.

## Methods

### Analysed samples

Between October 1995 and June 1996, all 170,457 junior high school students aged 11 to 16 years old (grades 7 to 9) in all 123 public and private junior high schools in the Kaohsiung and Pingtung areas in southern Taiwan were invited to participate a heath investigation [15]. The original purpose of the survey was to investigate the association between indoor and outdoor air pollution and health effects in adolescents. Of this sample, 165,173 adolescents completed questionnaires with a 96.9% response rate, 2,407 of whom were excluded who had an invalid national identity number and 84 of whom lacked age or sex data. Therefore, we designed a 12-years population-based cohort study and 162,682 participants were included in this analysis. This study was approved by the Institutional Review Board of the Committee on Human Subjects of Kaohsiung Medical University. All participants received a complete description of the original study and a written informed consent was provided from their parents or guardians on behalf of the children enrolled in our study.

### Measurements

Each student and his/her parents completed a structured questionnaire (including video instruction) concerning asthma symptom severity, allergic rhinitis, the habits of alcohol consumption and cigarette smoking. Items in the parental questionnaire included demographic characteristics (sex, age, parental highest education level), family smoking status (how many family members living with the student were regular smokers), the total amount of cigarettes per day used by the family, the student’s exercise habits (none, seldom, usual), and a history in the student of allergic rhinitis. SHS was defined on the basis of at least one family member living with the student who was a regular smoker, with the total daily cigarette consumption by family members calculated. We grouped SHS status into three groups of no, 1–20 cigarettes and >20 cigarettes exposure. Information on students’ cigarette smoking and alcohol consumption was obtained from the students themselves and the asthma status and symptom profile of each student were obtained through a video guided questionnaire [16].

### Suicide and other cause mortality

**Fig 1:**
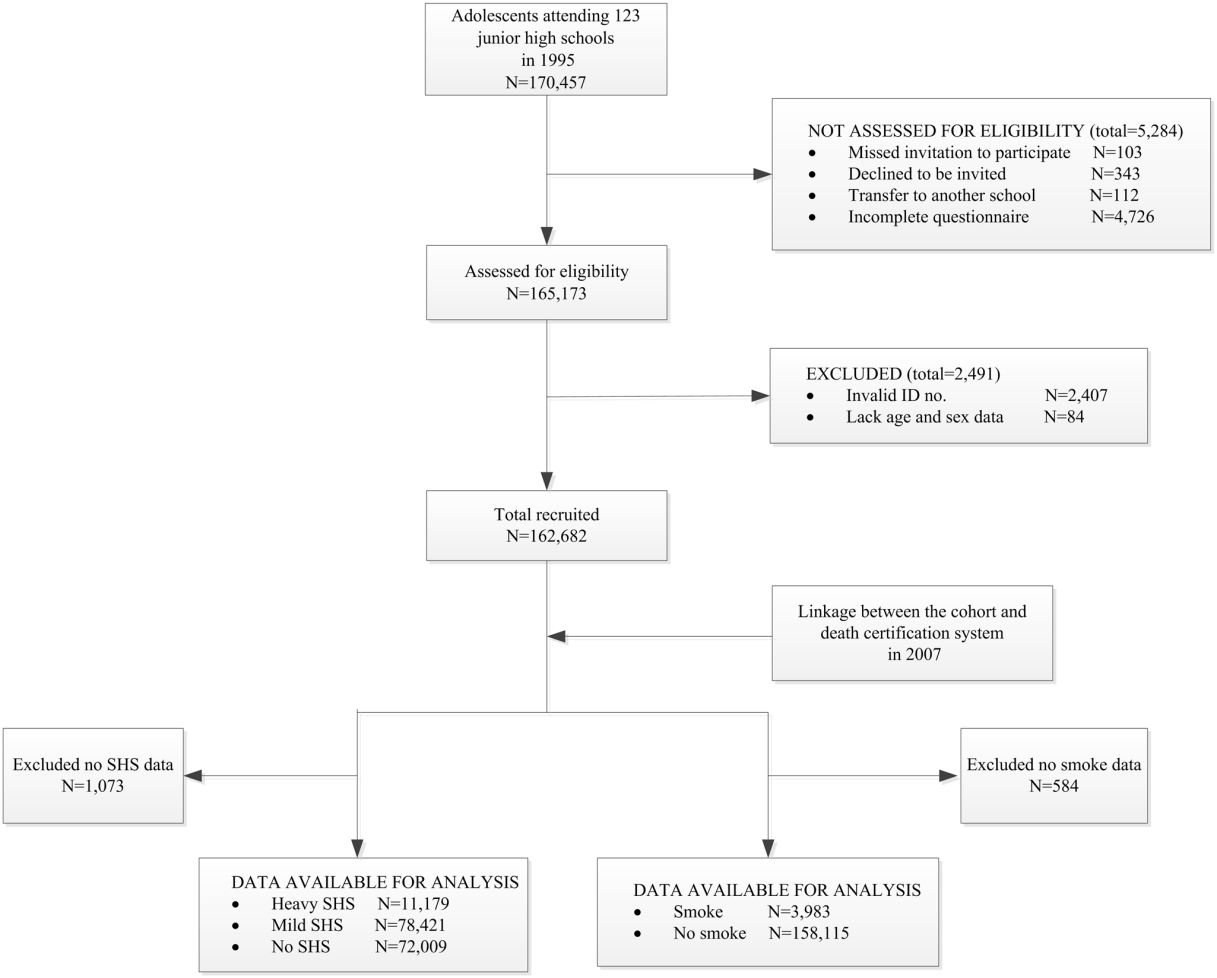
STROBE style flow chart of the cohort in southern Taiwan included in analysis.

The cohort was followed by record linkage to the Taiwan National Death Certification System from January 1, 1995, to December 31, 2007, as previously described [17]. Using unique and compulsory national identity numbers, linkage was made between the cohort and Death Certification System data which records causes of death. By December 31, 2007, 902 participants had died, with suicide listed as the cause of death for 106. The average and total duration of follow-up in this study was 11.98 years and 1,948,432.63 person-years (the flow chart as Fig 1).

### Statistical analysis

Mortality rates were calculated for specific causes in the smoking and nonsmoking groups, with lifetable survival analysis and Gehan’s generalized Wilcoxon tests used to assess differences in incidence. Cox proportional hazards models were used to investigate associations with smoking and SHS exposures, adjusting for gender, age, parental highest education, asthma, allergic rhinitis and alcohol consumption. Crude and adjusted hazard ratios and 95% confidence intervals were calculated with covariates chosen on the basis of significant or borderline association with suicide (p<0.15) in the unadjusted models. Population attributable fractions were calculated [18] using adjusted hazard ratios from final regression models.

### Sensitivity analyses

First, previous studies [19,20] have demonstrated a strong association between major depression in adolescence and suicide. In order to quantify the potential confounding effect of depression, Greenland’s method for sensitivity analysis was used to estimate the unmeasured confounding effect of depression. We performed a sensitivity analysis to control for this unmeasured potential confounding factor, using a well-established methodology [21] taking into account the known correlations between smoking and depression and between depression and suicide (details provided in S1 Text). Because previous studies had used odds ratios to measure the association between smoking and suicide, we also used the odds ratio as an index in these sensitivity analyses to approximate the hazard ratio.

Second, it was considered that some suicide deaths might have been misclassified as undetermined unnatural deaths [22]. Thus, we further combined undetermined unnatural death and suicide death as the outcome and estimated the risk of smoking and second hand smoking respectively to validate the associations.

Third, for obtaining unbiased proportional hazards regression coefficients, a prior study [23] had recommended modeling the events with age as the time-scale instead of time since baseline. Thus, we carried out additional analyses using age rather than time in the Cox models.

## Results

### Sample characteristics

A total of 162,682 students were recruited (response rate: 95%), 49.8% of whom were male. The mean (SD) age was 13.8 (0.9) years, smoking behavior was reported by 2.4%, and exposure to SHS of 1–20 cigarettes and >20 cigarettes were 48.5% and 6.9%, respectively (Table 1).

**Table 1:** Baseline sample characteristics (N = 162,682).

Characteristic	N* (%)
Age	
11–12 years	38712(23.8)
13–14 years	110061(67.6)
15–16 years	13909(8.5)
The amount of cigarette smoking by the family	
None	72009 (44.6)
1–20 cigarettes	78421(48.5)
>20 cigarettes	11179(6.9)
Cigarette smoking	
Absence	158115(97.1)
Presence	3983(2.4)
Alcohol drinking	
Absence	159158(97.8)
Presence	2799(1.7)
Asthma, lifetime	
Absence	132635(81.5)
Presence	30131(18.5)
Allergic rhinitis	
Absence	101580(62.4)
Presence	60953(37.4)

*Missing data excluded.

### Unadjusted associations between smoking / SHS exposure and mortality

Incidences of both natural and unnatural mortality were significantly higher in smokers compared to non-smokers (Table 2). Specifically, participants who were currently smoking at baseline had a greater than six-fold higher incidence rate of suicide mortality than those who did not smoke. Incidences of overall unnatural mortality, as well as suicide mortality specifically, were positively also associated with SHS exposure. Post hoc pairwise comparison found that the >20 cigarettes and 1–20 cigarettes SHS groups had higher incidences of suicide mortality than non-SHS group (12.7 and 5.8 versus 3.7 per 100,000 person-years, p<0.001, respectively) (Table 3).

**Table 2:** Cause-specific mortality by smoking status (N = 162,098).

Characteristics	Smoking (+) (N = 3,983)(PYs = 47533.57) ^a^		Smoking (-)(N = 158,115)(PYs = 1,900,899.06) ^a^		p
Cause of deaths	N. of deaths	Incidence^b^	N. of deaths	Incidence^b^	
Total mortality	82	172.5	820	43.1	<0.001
Unnatural death	66	138.8	624	32.8	<0.001
Accident	50	105.2	506	26.6	<0.001
Suicide	14	29.5	92	4.8	<0.001
Homicide	1	2.1	10	0.5	0.154
Undetermined for unnatural deaths	1	2.1	16	0.8	0.360
Natural death	16	33.7	196	10.3	<0.001
Cancer	1	2.1	74	3.9	0.536
Endocrine or Metabolic	1	2.1	8	0.4	0.093
Neurological	1	2.1	5	0.3	0.024
Cardiovascular	5	10.5	20	1.1	<0.001
Respiratory	1	2.1	7	0.4	0.066
Gastrointestinal	1	2.1	6	0.3	0.043
Genitourinary	0	0.0	6	0.3	0.699
Skin	0	0.0	8	0.4	0.656
Bone	0	0.0	6	0.3	0.699
Others	2	4.2	18	0.9	0.029
Undetermined for natural deaths	4	8.4	38	2.0	0.002

^a.^ PYs: person-years

^b.^ incidence: number per 100,000 person-years.

The difference of incidence in specific causes of deaths between smoker and non-smoker group were calculated using Wilcox (Gehan) Statistic by survival life table analysis.

**Table 3:** Incidence rates of causes of death among the subjects stratified by the status of second hand smoking (SHS) (N = 161,609).

	No SHS exposure		Mild SHS exposure		Heavy SHS exposure			Mild vs No	Heavy vs Mild	Heavy vs No
Characteristics	No (N = 72,009)(PYs = 866,081.34)		>0, < = 20 cigarettes (N = 78,421)(PYs = 942,235.54)		> 20 cigarettes(N = 11,179)(PYs = 134,227.33)		Overall p ^a^			
Cause of deaths	N. of deaths	Incidence	N. of deaths	Incidence	N. of deaths	Incidence				
Total mortality	324	37.4	480	50.9	91	67.8	<0.001***	<0.001***	0.011*	<0.001***
Unnatural death	240	27.7	367	38.9	77	57.4	<0.001***	<0.001***	0.002**	<0.001***
Accident	196	22.6	299	31.7	57	42.5	<0.001***	<0.001***	0.039*	<0.001***
Suicide	32	3.7	55	5.8	17	12.7	<0.001***	0.036*	0.004**	<0.001***
Homicide	4	0.5	6	0.6	1	0.7	0.850	0.617	0.883	0.666
Undetermined for unnatural deaths	8	0.9	7	0.7	2	1.5	0.674	0.674	0.376	0.541
Natural death	84	9.7	113	12.0	14	10.4	0.355	0.153	0.642	0.797
Cancer	35	4.0	36	3.8	3	2.2	0.608	0.815	0.365	0.318
Endocrine or Metabolic	5	0.6	4	0.4	0	0.0	0.639	0.644	0.451	0.379
Neurological	2	0.2	3	0.3	1	0.7	0.608	0.723	0.450	0.312
Cardiovascular	8	0.9	13	1.4	3	2.2	0.381	0.367	0.449	0.178
Respiratory	1	0.1	6	0.6	1	0.7	0.186	0.075	0.884	0.130
Gastrointestinal	4	0.5	3	0.3	0	0.0	0.678	0.624	0.514	0.431
Genitourinary	4	0.5	2	0.2	0	0.0	0.508	0.358	0.594	0.431
Skin	3	0.3	4	0.4	1	0.7	0.796	0.787	0.612	0.497
Bone	0	0.0	6	0.6	0	0.0	0.041*	0.019*	0.355	-
Others	8	0.9	12	1.3	1	0.7	0.716	0.476	0.604	0.843
Undetermined for natural deaths	14	1.6	24	2.5	4	3.0	0.358	0.209	0.714	0.267

^a^. testing by means of life tables analysis

*: p<0.05,

**: p<0.01,

***:p<0.001

### Adjusted associations with suicide mortality

The proportional hazards assumption over the entire follow-up period was confirmed for all estimated variables used in the Cox regression. The crude hazard ratio for suicide mortality in the smoking compared to non-smoking group was 6.1. After controlling for other covariates, the adjusted hazard ratio was 3.69 (Table 4). Family SHS also remained a significant predictor of suicide mortality in fully adjusted models with hazard ratios of 1.5 and 2.8 for 1–20 cigarettes and >20 cigarettes/per day, respectively. Despite males having a higher risk for suicide mortality than females in the analysis, there was no multiplicative interaction between family SHS and gender (p = 0.608) or between smoking and gender (p = 0.136) in associations with suicide. The suicide population attributable fraction for current smoking was 9.6%, and that for >20 cigarettes per day SHS was 10.6%.

**Table 4:** Cox proportional hazards regression analyses of factors associated with suicide mortality.

Characteristic	Associations with suicide mortality—hazard ratios (95% CI) displayed for simultaneously entered covariates				
	Unadjusted	Adjusted model 1^a^	Adjusted model 2 ^b^	Adjusted model 3 ^c^	Adjusted model 4 ^d^
Male/female	1.89 (1.27, 2.81)	1.88 (1.25, 2.82)	1.71 (1.13, 2.58)	1.64 (1.08, 2.47)	1.64 (1.09, 2.48)
Age, y	1.20 (0.97, 1.49)	1.15 (0.92, 1.44)	1.10 (0.88, 1.38)	1.13 (0.90, 1.41)	1.13 (0.90, 1.41)
The highest education of parents					
1 (primary school/illerate)	Reference	Reference	Reference	Reference	Reference
2 (high school)	0.97 (0.60, 1.55)	1.00 (0.62, 1.60)	1.03 (0.64, 1.66)	1.00 (0.62, 1.61)	1.00 (0.62, 1.60)
3 (college or higher)	0.85 (0.45, 1.61)	0.98 (0.50, 1.89)	1.04 (0.54, 2.03)	0.95 (0.49, 1.86)	0.95 (0.49, 1.86)
SHS (cigarettes)					
0	Reference	Reference	Reference	Reference	Reference
>0, < = 20	1.58 (1.02, 2.44)	1.55 (1.00, 2.41)	1.47 (0.94, 2.30)	1.47 (0.94, 2.30)	1.47 (0.94, 2.30)
>20	3.43 (1.91, 6.18)	3.36 (1.85, 6.10)	2.90 (1.58, 5.33)	2.82 (1.54, 5.18)	2.83 (1.54, 5.20)
Current smoking (yes/no)	6.11 (3.48, 10.72)	…	3.53 (1.87, 6.66)	3.33 (1.76, 6.28)	3.69 (1.85, 7.39)
Asthma, lifetime (yes/no)	2.36 (1.58, 3.52)	…	…	2.19 (1.45, 3.33)	2.20 (1.45, 3.34)
Allergic rhinitis (yes/no)	1.55 (1.06, 2.26)	…	…	1.30 (0.87, 1.94)	1.30 (0.87, 1.94)
Alcoholic drinking (yes/no)	2.25 (0.83, 6.11)	…	…	…	0.70 (0.23, 2.11)

Note. HR = hazard ratio; CI = confidence interval; AHR = adjusted hazard ratio

^a^Adjusted for gender, age, SHS exposure and the highest education of parents,

^b^Adjusted for gender, age, the highest education of parents, SHS exposure and cigarette smoking,

^c^Adjusted for gender, age, the highest education of parents, SHS exposure, cigarette smoking, the lifetime asthma, and allergic rhinitis,

^d^Adjusted for gender, age, the highest education of parents, SHS exposure, cigarette smoking, the lifetime asthma, allergic rhinitis, and alcoholic drinking

### Sensitivity analyses

In sensitivity analyses, following further external adjustment for depression as an unmeasured confounder, the odds ratio between smoking and suicide was 4.80 (see S1 Text), comparable in strength to that derived from the main analyses.

Combining suicide and undetermined unnatural death as the outcome, the adjusted hazard ratio for suicide mortality in the smoking compared to non-smoking group was 3.81, which was equivalent to the effect size from the main analysis (3.69), as was that for family SHS (S1 Table). Finally, substitution of age for time since baseline in the regression models did not substantially alter findings (S2 Table).

## Discussion

In a large prospective cohort study, assembled using data linkage, we investigated the association of active and passive smoking exposure with suicide specifically in the context of other-cause mortality more widely. To our knowledge, ours is the first study to extend previous findings potentially linking smoking and suicide to an investigation in adolescents specifically. Additionally, we believe ours to be the first investigation of the association between SHS exposure and suicide. Both smoking and SHS exposure were found to be independently associated with increased risk of suicide, findings for the latter indicating a dose-response relationship.

Previous studies of smoking and suicide have reported inconsistent findings. Some epidemiological studies have found an independent association [6,9,24], whereas others studies have found that this did not persist when confounders were taken into consideration [25,26]. However, it remains unclear whether some of the adjustments made are for confounding or causal pathway factors: for example, smoking predicting or giving rise to mental disorders, which in turn affect risk of suicide [5]. A recent meta-analysis synthesized available data from fifteen prospective cohort studies involving 2395 deaths among 1,369,807 participants, and reported pooled risk ratios for suicide of 1.28 for former smokers, and 1.81 for current smokers compared with never smokers [4].

A range of possible explanations for the association between smoking and suicide have been proposed. To begin with, smoking may be a non-causal marker of other risk factors for suicide such as lower income, lower education, unmarried status, unemployment, or lack of religious affiliation. It may also be a marker for mental disorders such as anxiety, depression, psychoses, and substance use disorders, or indicate other adverse mental health states such as low self-esteem, a propensity to risk-taking behaviour, impulsivity, aggression, antisocial personality, fatalism, or emotional instability. People with pre-existing or “latent” psychiatric or psychological problems may use nicotine to “self-medicate” to abate these problems [5,27]. However, there may also be direct links between smoking and suicide. Specifically, chronic nicotine exposure reduces serotonin and its metabolites in animals [28], and smokers have been found to have lower cerebrospinal fluid levels of serotonin metabolites [29], and lower levels of platelet monoamine oxidase [30]. Smoking also clearly causes a range of physical illnesses including chronic respiratory disease, cardiovascular disease and cancer, and worse physical health is recognized to be a leading cause of suicide [17,31,32]; however, this causal pathway might be expected to be less salient in adolescents than older age groups. Although mental disorders are a well-known risk factor for suicide, such conditions are often unrecognized, even in medical settings. Therefore, regardless of whether it is a ‘risk factor’ or ‘risk marker’, smoking may be a useful clinical indicator of a risk for subsequent suicide [9].

Some large prospective studies have controlled for alcohol consumption, and continued to find an association between smoking and suicide [24,33]; but another did not [34]. The present study found that the association of interest persisted after adjustment for alcohol use, although measurement of this was restricted to consumption or not. It should also be borne in mind that our cohort may have been too young for alcohol to have exerted an independent effect, since most alcohol-related suicide is related to excessive consumption over much longer periods [35]. One recent study has found an association between asthma and higher suicide risk [17], although in our study the associations of smoking and SHS with suicide were independent of asthma.

We found an association between SHS exposure and suicide in adolescents which, to our knowledge, has not been previously investigated. Clearly the findings require replication, although the association is plausible. In non-smoking adolescents, duration of exposure to SHS in the home was found to have a positive dose-response relationship with psychological distress as measured by the 12-item General Health Questionnaire [36]. Adolescent current smokers who are exposed to SHS at home have also been found to be more likely to report respiratory symptoms compared to those unexposed [12]. SHS is causally linked to an increased incidence and severity of childhood and adult asthma, lower respiratory tract infections, respiratory symptoms, otitis media, cancer and cardiovascular disease [37], and associations with suicide may in part reflect some of these health consequences.

### Strengths and limitations

Key strengths of the study were the large, representative sample and comprehensive follow-up. Limitations primarily reflect the use of historical data collected for purposes other than the objectives of this analysis. The information from questionnaire included measures of demographic characteristics, whether alcohol was consumed or not, smoking status and SHS exposure, although these relied on self-reporting and may be subject to measurement error. More importantly, information was lacking on certain risk factors for suicide such as mental disorders, personality traits, illicit drug use and family history of mental disorders. Although, a sensitivity analysis did not suggest that depression was likely to be a major confounding factor, the influence of mental health on the associations of interest could not be comprehensively evaluated. Besides mental disorders, other factors such as parental divorce, low emotional control, medication for nervous problems, contact with police and childcare services, drug use, and education may confound associations between smoking and suicide [25]. Because of the relatively large change in the hazard ratio (from 6.11 to 3.69) following adjustment, residual confounding is an important consideration and interpretation of the association between smoking exposure (direct or secondhand) and suicide should be cautious. The results also showed a strong association between smoking/second hand smoking and accidental deaths, which might also reflect common underlying social and behavioural/psychological causes. One previous study in Taiwan also reported an association between smoking and accident death, although was only able to adjust for education and alcohol use [10]. Furthermore, adolescents exposed to SHS might have been more likely to have parents with mental disorders. However, the SHS effect was not affected substantially by adjustment for other factors (HR changing from 3.43 to 2.83); whether the hypothesized SHS effect is more stable than that of smoking warrants further research. Finally, quantification of physical health was limited to specific disorders, although in this age group its influence would not be expected to be prominent.

Despite the relatively low smoking prevalence (2.4%) in our cohort, the prospect of longer-term exposure and consequent adverse outcomes are important issues for adolescent smoking. Furthermore, more than half of the adolescent population had been involuntarily exposed to SHS in this 1995 initiative cohort study. Protecting younger populations from early life cigarette smoking exposure is an important issue and needs a public health approach. Initial contact advice and reference materials provided by the pediatrician, followed by frequent, regular contacts and home visits was found to be an ideal approach [38], as well as involvement of school teachers or mentors, and smoke-free legislation [39], which has been implemented since 2009 in Taiwan. In addition, clinical staff should be reminded of the need for awareness of, and prevention measures to improve, mental health and suicide risk in young people with smoking and SHS exposure. This study found evidence of excess suicide among adolescents with active and passive early life cigarette smoke exposure which adds yet another adverse outcome to an already at-risk group. The associations between smoking, SHS and suicide were independent of each other and were not fully explained by gender, age, asthma, allergic rhinitis, alcohol use, or depression. However, further exploration of underlying causal relationships is needed. For example, the effect of SHS on the level of serotonin, other medical disorders or mental disorders and consequent suicide risks warrants more studies to investigate.

## Supporting Information

S1 Table Cox proportional hazards regression analyses of factors associated with combined suicide and undetermined-cause unnatural mortality. (DOCX)

S2 Table Cox proportional hazards regression analyses of factors associated with suicide mortality using age rather than time since baseline, as suggested by Korn et al (1997). (DOCX)

S3 Table STROBE Statement—checklist of items that should be included in reports of our cohort study. (DOCX)

S1 Text Sensitivity Analysis. (DOCX)

## References

[1] General S. Preventing tobacco use among youth and young adults. Atlanta, GA: Department of Health and Human Services, Centers for Disease Control and Prevention 2012; 12. 22876391

[2] Jha P, Ranson MK, Nguyen SN, Yach D. Estimates of global and regional smoking prevalence in 1995, by age and sex. *American journal of public health* 2002; 92: 1002–1006. 12036796 10.2105/ajph.92.6.1002 PMC1447501

[3] Wipfli H, Samet J. Global economic and health benefits of tobacco control: part 2. *Clinical Pharmacology & Therapeutics* 2009; 86: 272–280. 19536066 10.1038/clpt.2009.94

[4] Li D, Yang X, Ge Z, Hao Y, Wang Q, Liu F, et al Cigarette smoking and risk of completed suicide: a meta-analysis of prospective cohort studies. *Journal of psychiatric research* 2012; 46: 1257–1266. 22889465 10.1016/j.jpsychires.2012.03.013

[5] Hughes JR. Smoking and suicide: a brief overview. *Drug and alcohol dependence* 2008; 98: 169–178. 18676099 10.1016/j.drugalcdep.2008.06.003 PMC2585177

[6] Miller M, Hemenway D, Bell NS, Yore MM, Amoroso PJ. Cigarette smoking and suicide: a prospective study of 300,000 male active-duty Army soldiers. *American journal of epidemiology* 2000; 151: 1060–1063. 10873129 10.1093/oxfordjournals.aje.a010148

[7] Breslau N, Schultz LR, Johnson EO, Peterson EL, Davis GC. Smoking and the risk of suicidal behavior: a prospective study of a community sample. Archives of General Psychiatry 2005; 62: 328–334. 15753246 10.1001/archpsyc.62.3.328

[8] Doll R, Peto R, Wheatley K, Gray R, Sutherland I. Mortality in relation to smoking: 40 years' observations on male British doctors. *Bmj* 1994; 309: 901–911. 7755693 10.1136/bmj.309.6959.901 PMC2541142

[9] Iwasaki M, Akechi T, Uchitomi Y, Tsugane S. Cigarette smoking and completed suicide among middle-aged men: a population-based cohort study in Japan. Annals of epidemiology 2005; 15: 286–292. 15780776 10.1016/j.annepidem.2004.08.011

[10] Wen C, Tsai S, Cheng T, Chan H, Chung W, Chen C. Excess injury mortality among smokers: a neglected tobacco hazard. Tobacco control 2005; 14: i28–i32. 15923444 10.1136/tc.2003.005629 PMC1766175

[11] Riala K, Alaräisänen A, Taanila A, Hakko H, Timonen M, Räsänen P. Regular daily smoking among 14-year-old adolescents increases the subsequent risk for suicide: the Northern Finland 1966 Birth Cohort Study. *The Journal of clinical psychiatry* 2007; 68: 775–780. 17503989 10.4088/jcp.v68n0518

[12] Lai H-K, Ho S-Y, Wang M-P, Lam T-H. Secondhand smoke and respiratory symptoms among adolescent current smokers. *Pediatrics* 2009; 124: 1306–1310. 19841127 10.1542/peds.2009-0669

[13] Hamer M, Stamatakis E, Batty GD. Objectively assessed secondhand smoke exposure and mental health in adults: cross-sectional and prospective evidence from the Scottish Health Survey. Archives of general psychiatry 2010; 67: 850–855. 10.1001/archgenpsychiatry.2010.76 20529994

[14] Bandiera FC, Richardson AK, Lee DJ, He J-P, Merikangas KR. Secondhand smoke exposure and mental health among children and adolescents. *Archives of pediatrics & adolescent medicine* 2011; 165: 332–338. 21464381 10.1001/archpediatrics.2011.30 PMC3075798

[15] Wang T-N, Ko Y-C, Chao Y-Y, Huang C-C, Lin R-S. Association between indoor and outdoor air pollution and adolescent asthma from 1995 to 1996 in Taiwan. *Environmental research* 1999; 81: 239–247. 10585020 10.1006/enrs.1999.3985

[16] Shaw R, Crane J, O'Donnell T, Lewis M, Stewart B, Beasley R. The use of a videotaped questionnaire for studying asthma prevalence. A pilot study among New Zealand adolescents. *The Medical journal of Australia* 1992; 157: 311–314. 1435471

[17] Kuo C-J, Chen VC-H, Lee W-C, Chen WJ, Ferri CP, Stewart R, et al Asthma and suicide mortality in young people: a 12-year follow-up study. *American journal of psychiatry* 2010; 167: 1092–1099. 20634368 10.1176/appi.ajp.2010.09101455

[18] Greenland S. Applications of stratified analysis methods Modern Epidemiology 3rd ed.; 2008.

[19] Hallfors DD, Waller MW, Ford CA, Halpern CT, Brodish PH, Iritani B. Adolescent depression and suicide risk: association with sex and drug behavior. *American journal of preventive medicine* 2004; 27: 224–231. 15450635 10.1016/j.amepre.2004.06.001

[20] Gould MS, Greenberg T, Velting DM, Shaffer D. Youth suicide risk and preventive interventions: a review of the past 10 years. Journal of the American Academy of Child & Adolescent Psychiatry 2003; 42: 386–405. 12649626 10.1097/01.CHI.0000046821.95464.CF

[21] Rothman KJ, Greenland S, Lash TL. Modern epidemiology. Lippincott Williams & Wilkins; 2008.

[22] Chang S-S, Sterne JA, Lu T-H, Gunnell D. ‘Hidden’suicides amongst deaths certified as undetermined intent, accident by pesticide poisoning and accident by suffocation in Taiwan. Social psychiatry and psychiatric epidemiology 2010; 45: 143–152. 10.1007/s00127-009-0049-x 19363577

[23] Kom EL, Graubard BI, Midthune D. Time-to-event analysis of longitudinal follow-up of a survey: choice of the time-scale. *American journal of epidemiology* 1997; 145: 72–80. 8982025 10.1093/oxfordjournals.aje.a009034

[24] Miller M, Hemenway D, Rimm E. Cigarettes and suicide: a prospective study of 50,000 men. *American journal of public health* 2000; 90: 768. 10800427 10.2105/ajph.90.5.768 PMC1446219

[25] Hemmingsson T, Kriebel D. Smoking at age 18–20 and suicide during 26 years of follow-up—how can the association be explained? *International journal of epidemiology* 2003; 32: 1000–1004. 14681264 10.1093/ije/dyg203

[26] Boden JM, Fergusson DM, Horwood LJ. Cigarette smoking and suicidal behaviour: results from a 25-year longitudinal study. Psychological Medicine 2008; 38: 433 17892622 10.1017/S0033291707001547

[27] Markou A, Kenny PJ. Neuroadaptations to chronic exposure to drugs of abuse: relevance to depressive symptomatology seen across psychiatric diagnostic categories. Neurotoxicity research 2002; 4: 297–313. 12829420 10.1080/10298420290023963

[28] Olausson P, Engel JA, Söderpalm B. Involvement of serotonin in nicotine dependence: processes relevant to positive and negative regulation of drug intake. *Pharmacology Biochemistry and Behavior* 2002; 71: 757–771. 10.1016/s0091-3057(01)00673-6 11888567

[29] Malone KM, Waternaux C, Haas GL, Cooper TB, Li S, Mann JJ. Cigarette smoking, suicidal behavior, and serotonin function in major psychiatric disorders. *American Journal of Psychiatry* 2003. 10.1176/appi.ajp.160.4.773 12668368

[30] Lewitzka U, Müller‐Oerlinghausen B, Felber W, Brunner J, Hawellek B, Rujescu D, et al Is MAO‐B activity in platelets associated with the occurrence of suicidality and behavioural personality traits in depressed patients? *Acta Psychiatrica Scandinavica* 2008; 117: 41–49. 18028252 10.1111/j.1600-0447.2007.01121.x

[31] Sudak H. Kaplan & Sadock's Comprehensive Testbook of Psychiatry; Edition t, editor. New York:. Lippincott Williams and Wilkins: Psychiatric emergencies; 1999.

[32] Crump C, Sundquist K, Sundquist J, Winkleby M. Sociodemographic, psychiatric and somatic risk factors for suicide: a Swedish national cohort study. Psychological medicine 2014; 44: 279–289. 10.1017/S0033291713000810 23611178

[33] Paffenbarger RS, Lee IM, Leung R. Physical activity and personal characteristics associated with depression and suicide in American college men. *Acta Psychiatrica Scandinavica* 1994; 89: 16–22. 10.1111/j.1600-0447.1994.tb05796.x 8053361

[34] Klatsky AL, Armstrong MA. Alcohol use, other traits, and risk of unnatural death: a prospective study. *Alcoholism*: *Clinical and Experimental Research* 1993; 17: 1156–1162. 10.1111/j.1530-0277.1993.tb05221.x 8116824

[35] DiFranza JR, Guerrera MP. Alcoholism and smoking. *Journal of studies on alcohol* 1990; 51: 130–135. 2308350 10.15288/jsa.1990.51.130

[36] Padrón A, Galán I, Rodríguez-Artalejo F. Second-hand smoke exposure and psychological distress in adolescents. A population-based study. Tobacco control 2014; 23: 302 10.1136/tobaccocontrol-2012-050548 23002180

[37] Lounsbery MG, Bubak ME. The impact of secondhand smoke on children: respiratory and other medical concerns. South Dakota medicine: the journal of the South Dakota State Medical Association 2008: 13–16. 19363888

[38] Polanska K, Hanke W, Ronchetti R, HAZEL P, Zuurbier M, Koppe JG, et al Environmental tobacco smoke exposure and children's health. *Acta paediatrica* 2006; 95: 86–92. 10.1080/08035320600886562 17000575

[39] Mackay D, Haw S, Ayres JG, Fischbacher C, Pell JP. Smoke-free legislation and hospitalizations for childhood asthma. *New England Journal of Medicine* 2010; 363: 1139–1145. 20843248 10.1056/NEJMoa1002861

