# Supplementary material for: Suicide and Other-Cause Mortality after Early Exposure to Smoking and Second Hand Smoking: A 12-Year Population-Based Follow-Up Study
Source: PLoS One. 2015 Jul 29;10(7):e0130044. doi: 10.1371/journal.pone.0130044 (PMC4519334; doi:10.1371/journal.pone.0130044)
Supplement: S1 Table — (DOCX) [file pone.0130044.s001.docx]

**Supporting Information**

S1 Table. Cox proportional hazards regression analyses of factors associated with combined suicide and undetermined-cause unnatural mortality

| Characteristic | Associations with suicide/undetermined mortality – hazard ratios (95% CI) displayed for simultaneously entered covariates | | | | |
| --- | --- | --- | --- | --- | --- |
|  | Unadjusted | Adjusted model 1^a^ | Adjusted model 2 ^b^ | Adjusted model 3 ^c^ | Adjusted model 4 ^d^ |
| Male/female | 1.64 (1.14, 2.35) | 1.63 (1.12, 2.35) | 1.48 (1.02, 2.16) | 1.44 (0.99, 2.09) | 1.44 (0.99, 2.10) |
| Age, y | 1.14 (0.93, 1.39) | 1.10 (0.89, 1.35) | 1.05 (0.86, 1.29) | 1.07 (0.87, 1.31) | 1.07 (0.87, 1.31) |
| The highest education of parents |  |  |  |  |  |
| 1 (primary school/illerate) | Reference | Reference | Reference | Reference | Reference |
| 2 (high school) | 1.01 (0.65, 1.58) | 1.03 (0.66, 1.61) | 1.07 (.68, 1.67) | 1.04 (0.67, 1.63) | 1.04 (0.66, 1.63) |
| 3 (college or higher) | 0.85 (0.47, 1.55) | 0.94 (0.51, 1.76) | 1.00 (0.54, 1.87) | 0.94 (0.50, 1.76) | 0.94 (0.50, 1.76) |
| SHS (cigarettes) |  |  |  |  |  |
| 0 | Reference | Reference | Reference | Reference | Reference |
| >0, <=20 | 1.43 (0.96, 2.12) | 1.39 (0.93, 2.08) | 1.33 (0.88, 1.99) | 1.33 (0.88, 1.99) | 1.33 (0.88, 1.99) |
| >20 | 3.07 (1.78, 5.30) | 3.00 (1.73, 5.21) | 2.61 (1.49, 4.58) | 2.55 (1.45, 4.48) | 2.56 (1.46, 4.50) |
| Current smoking (yes/no) | 5.57 (3.25, 9.56) |  | 3.52 (1.92, 6.46) | 3.34 (1.82, 6.13) | 3.81 (1.97, 7.36) |
| Asthma, lifetime (yes/no) | 2.12 (1.46, 3.10) | … | … | 2.04 (1.38, 3.02) | 2.05 (1.38, 3.03) |
| Allergic rhinitis (yes/no) | 1.31 (0.91, 1.86) | … | … | 1.12 (0.77, 1.62) | 1.12 (0.77, 1.62) |
| Alcoholic drinking (yes/no) | 1.93 (0.71, 5.23) | … | … | … | 0.63 (0.21, 1.88) |

Note. HR=hazard ratio; CI=confidence interval; AHR=adjusted hazard ratio

^a^Adjusted for gender, age, SHS exposure and the highest education of parents, ^b^Adjusted for gender, age, the highest education of parents, SHS exposure and cigarette smoking, ^c^Adjusted for gender, age, the highest education of parents, SHS exposure, cigarette smoking, the lifetime asthma, and allergic rhinitis, ^d^Adjusted for gender, age, the highest education of parents, SHS exposure, cigarette smoking, the lifetime asthma, allergic rhinitis, and alcoholic drinking, *p<0.05, **p<0.01, ***p<0.001
