# Supplementary material for: Suicide and Other-Cause Mortality after Early Exposure to Smoking and Second Hand Smoking: A 12-Year Population-Based Follow-Up Study
Source: PLoS One. 2015 Jul 29;10(7):e0130044. doi: 10.1371/journal.pone.0130044 (PMC4519334; doi:10.1371/journal.pone.0130044)
Supplement: S2 Table — (DOCX) [file pone.0130044.s002.docx]

**Supporting Information**

S2 Table. Cox proportional hazards regression analyses of factors associated with suicide mortality using age rather than time since baseline, as suggested by Korn et al (1997)[[5](#_ENREF_5)].

| Characteristic | Associations with suicide mortality – hazard ratios (95% CI) displayed for simultaneously entered covariates | | | | |
| --- | --- | --- | --- | --- | --- |
|  | Unadjusted | Adjusted model 1^a^ | Adjusted model 2 ^b^ | Adjusted model 3 ^c^ | Adjusted model 4 ^d^ |
| Male/female | 1.89 (1.27, 2.81) | 1.88 (1.25, 2.82) | 1.71 (1.13, 2.58) | 1.63 (1.08, 2.47) | 1.64 (1.08, 2.48) |
| Age, y | 1.06 (0.84, 1.33) | 1.01 (0.80, 1.27) | 0.96 (0.76, 1.22) | 0.98 (0.78, 1.24) | 0.98 (0.78, 1.24) |
| The highest education of parents |  |  |  |  |  |
| 1 (primary school/illerate) | Reference | Reference | Reference | Reference | Reference |
| 2 (high school) | 0.99 (0.61, 1.59) | 1.00 (0.62, 1.60) | 1.03 (0.64, 1.66) | 1.00 (0.62, 1.61) | 1.00 (0.62, 1.61) |
| 3 (college or higher) | 0.88 (0.47, 1.67) | 0.98 (0.50, 1.89) | 1.05 (0.54, 2.03) | 0.96 (0.49, 1.86) | 0.95 (0.49, 1.86) |
| SHS (cigarettes) |  |  |  |  |  |
| 0 | Reference | Reference | Reference | Reference | Reference |
| >0, <=20 | 1.58 (1.02, 2.44) | 1.55 (1.00, 2.41) | 1.47 (0.94, 2.30) | 1.47 (0.94, 2.30) | 1.47 (0.94, 2.30) |
| >20 | 3.40 (1.89, 6.12) | 3.36 (1.85, 6.10) | 2.91 (1.58, 5.33) | 2.82 (1.54, 5.18) | 2.84 (1.55, 5.20) |
| Current smoking (yes/no) | 5.80 (3.31, 10.19) |  | 3.54 (1.88, 6.68) | 3.34 (1.77, 6.30) | 3.71 (1.86, 7.42) |
| Asthma, lifetime (yes/no) | 2.39 (1.60, 3.56) |  |  | 2.19 (1.45, 3.32) | 2.20 (1.45, 3.33) |
| Allergic rhinitis (yes/no) | 1.55 (1.06, 2.27) |  |  | 1.30 (0.87, 1.94) | 1.30 (0.87, 1.94) |
| Alcoholic drinking (yes/no) | 2.19 (0.81, 5.94) |  |  |  | 0.70 (0.23, 2.11) |

Note. HR=hazard ratio; CI=confidence interval; AHR=adjusted hazard ratio

^a^Adjusted for gender, age, SHS exposure and the highest education of parents, ^b^Adjusted for gender, age, the highest education of parents, SHS exposure and cigarette smoking, ^c^Adjusted for gender, age, the highest education of parents, SHS exposure, cigarette smoking, the lifetime asthma, and allergic rhinitis, ^d^Adjusted for gender, age, the highest education of parents, SHS exposure, cigarette smoking, the lifetime asthma, allergic rhinitis, and alcoholic drinking, *p<0.05, **p<0.01, ***p<0.001
