# Supplementary material for: Suicide and Other-Cause Mortality after Early Exposure to Smoking and Second Hand Smoking: A 12-Year Population-Based Follow-Up Study
Source: PLoS One. 2015 Jul 29;10(7):e0130044. doi: 10.1371/journal.pone.0130044 (PMC4519334; doi:10.1371/journal.pone.0130044)
Supplement: S1 Text — (DOCX) [file pone.0130044.s004.docx]

**Supporting Information**

**Suicide and other-cause mortality after early exposure to smoking and second hand smoking: a 12-Year population-based follow-up study**

Vincent Chin-Hung Chen, Chian-Jue Kuo, Tsu-Nai Wang, Wen-Chung Lee, Wei-J Chen , Cleusa P Ferri, Duujian Tsai, Te-Jen Lai, Meng-Chuan Huang, Robert Stewart, Ying-Chin Ko

**S1 Text. Sensitivity Analysis**

The method of Greenland et al [[1](#_ENREF_1)] was used to quantify the confounding effect of depression on our estimates of the association between smoking and suicide mortality based on previous studies of depression and suicide among youth [[2](#_ENREF_2), [3](#_ENREF_3)].

The prevalences of depression in the smoking group and non-smoking group as well as the odds ratio for the association between depression and suicide among young people were inferred from previous studies for external adjustment in the sensitivity analysis. A previous study [[2](#_ENREF_2)] of subjects with ages similar to those in our study showed the prevalence of depression in a smoking group to be 30.6%, while in a non-smoking group, the prevalence was 19.8%. Another study [[3](#_ENREF_3)] estimated the depression-suicide odds ratio among youth with ages similar to those of our study was 5.69.

The crude odds ratio of suicide in the smoking group with relative to the non-smoking group was 6.06, and after external adjustment a depression-adjusted odds ratio of 4.80 was obtained with little change.

In addition, we adopted the prevalence of depression in adolescents from a Taiwan study [[4](#_ENREF_4)] which had used the Chinese version of the Center for Epidemiologic Studies Depression Scale (CES-D) and calculated the depression-adjusted odds ratio. Reported prevalences of mild or severe depression in smoking and non-smoking groups were 43.2% and 28.9% respectively. After external adjustment, a depression-adjusted odds ratio was 4.72 with relatively little change (as compared with 6.06). Substituting prevalences of severe depression in smoking and non-smoking groups (15.5% and 11.8% respectively) the depression-adjusted odds ratio was 5.45, also little changed.

Further analysis was performed using an extreme hypothetical scenario with depression prevalence of 40% for the smoking group and 20% for the non-smoking group, and a depression-suicide odds ratio of 10. The depression-adjusted odds ratio for the association between smoking and suicide mortality was 3.69, without changing the direction of the association.

**References**

1. Greenland S, Lash TL. Bias analysis. In: Rothman KJ, Greenland S, Lash TL, editors. Modern Epidemiology, Third ed. Philadelphia: Lippincott Williams & Wilkins; 2008. p. 348-351.

2. Katon W, Richardson L, Russo J, McCarty CA, Rockhill C, McCauley E, et al. Depressive symptoms in adolescence: the association with multiple health risk behaviors. *Gen Hosp Psychiatry* 2010; 32: 233-239.

3. Hallfors DD, Waller MW, Ford CA, Halpern CT, Brodish PH, Iritani B. Adolescent depression and suicide risk: association with sex and drug behavior. *Am J Prev Med* 2004; 27: 224-231.

4. Wang PW, Lin HC, Yeh YC, Liu TL, Yen CF. The relation of substance use with different levels of depressive symptoms and the moderating effect of sex and age in Taiwanese adolescents. *Compr Psychiatry* 2012; 53: 1013-1020.
